# 14/w – „schlechte“ Haltung und leicht abstehendes Schulterblatt: Vorbereitung auf die Facharztprüfung: Fall 47

**DOI:** 10.1007/s00132-020-04038-9

**Published:** 2020-11-19

**Authors:** Alexander Krenauer, Christoph Mehren

**Affiliations:** Schön Klinik München Harlaching, Wirbelsäulenzentrum, Akademisches Lehrkrankenhaus der Paracelsus Medizinischen Universität Salzburg, Harlachinger Straße 51, 81547 München, Deutschland

**Keywords:** Cobb-Winkel, Lenke-Klassifikation, Risser-Zeichen, Skoliose, Wirbelsäule

## Prüfungssimulation

### Fallschilderung

Die 14-jährige Michaela stellt sich in Begleitung ihrer Eltern in der orthopädischen Sprechstunde vor. Die Eltern berichten, dass ihnen die schlechte Haltung ihrer Tochter aufgefallen sei. Zusätzlich würde ein Schulterblatt leicht abstehen und sie hätten den Eindruck, dass die Schultern und das Becken „schief“ stehen würden. Neuerdings klage ihre Tochter auch bei längerer Belastung über Rückenschmerzen. Ein Trauma habe nicht vorgelegen. Grundsätzlich sei ihre Tochter sehr aktiv und treibe regelmäßig Sport. Vorerkrankungen habe ihre Tochter keine.

## Prüfungsfragen

- Welche klinischen Untersuchungen führen Sie durch und welche weiteren Fragen erheben sie anamnestisch?
- Welche Diagnostik ist nötig?
- Was werten Sie auf dem Röntgenbild aus?
- Nach welchem Klassifikationssystem erfolgt die Einteilung der idiopathischen Skoliose?
- Welche Therapie leiten Sie ein und nach welchen Kriterien bestimmen Sie die Therapie?
- Wann würden Sie die Strategie einer konservativen Therapie ändern?
- Nennen Sie 2 weitere Formen von Skoliosen.

### Antworten

#### Welche klinischen Untersuchungen führen Sie durch und welche weiteren Fragen erheben sie anamnestisch?

##### Anamnese

- Vorerkrankungen
- Familienanamnese bezüglich Wirbelsäulenerkrankungen
- Bei weiblichen Patienten: Menarche bereits vorhanden?

##### Klinische Untersuchung

- Die klinische Untersuchung erfolgt am entkleideten Patienten.
- Beurteilung in der Frontalebene: lotgerechte Stellung der Wirbelsäule, Taillendreiecke, Schulter- und Beckenstellung, Stellung der Schulterblätter
- seitliche Ansicht: Seitprofil zur Beurteilung einer vermehrten oder abgeflachten thorakalen Kyphose
- Abklopfen und Abtasten der Wirbelsäule: Schmerzsymptomatik, myofasziale Triggerpunkte
- Untersuchung der Iliosakralgelenke
- Beurteilung der Inklination und Reklination der Wirbelsäule, besteht eine Limitierung der Seitneigung?
- Adam-Test: Beurteilung der Ausbildung eines Lendenwulstes und/oder eines Rippenbuckels
- Finger-Boden-Abstand
- im Liegen Beurteilung der Hüft-Lenden-Strecksteife
- orientierende neurologische Untersuchung

##### Der Fall.

Es zeigt sich eine Lotabweichung der Wirbelsäule nach links. Die Taillendreiecke sind asymmetrisch mit einer linksseitigen Betonung. Schiefstellung der Schulter mit Hochstand rechtsseitig. Angedeuteter Beckenschiefstand mit Hochstand rechtsseitig. Asymmetrische Stellung der Schulterblätter. Abgeflachte thorakale Kyphose. Es liegt ein paravertebraler Muskelhartspann vor, welcher im Bereich der Brustwirbelsäule rechtsseitig und der Lendenwirbelsäule linksseitig leicht druckschmerzhaft ist. Es zeigt sich kein Druckschmerz im Bereich der Iliosakralgelenke. In Inklination und Reklination werden keine Schmerzen angegeben. Es liegt keine Limitierung der Seitneigung vor. In Vorneigung zeigen sich ein ausgeprägter Rippenbuckel rechts und ein ausgeprägter Lendenwulst links. Der Finger-Boden-Abstand beträgt 0 cm und grobneurologisch zeigen sich keine Auffälligkeiten. Es besteht keine Hüft-Lenden-Strecksteife.

Aus der Familienanamnese ergibt sich, dass sowohl die Großmutter väterlicherseits, als auch die Mutter eine idiopathische Skoliose haben.

Die erste Regelblutung sei bei der Patientin vor ca. 2 Jahren (mit 12 Jahren) aufgetreten. Weitere Vorerkrankungen seien nicht bekannt.

#### Welche Diagnostik ist nötig?

- Als erster Schritt: **Röntgen** der gesamten Wirbelsäule **in 2 Ebenen**
- Bei Vorliegen einer **idiopathischen Skoliose**: Zur weiteren Beurteilung und Klassifizierung ist eine **Bendingaufnahme** nach links und nach rechts notwendig (→ Beurteilung der Rigidität).
- CT und MRT sind nur für spezielle Fragestellungen sinnvoll.
- EOS®-System: Eine moderne Bildgebung in spezialisierten Zentren; Möglichkeit der exakten 3D-Beurteilung der Skoliose mit deutlich verminderter Strahlenbelastung.

##### Cave

Mehrere Kohortenstudien ergaben im langfristigen Verlauf erhöhte Raten von u. a. Mammakarzinomen bei Skoliosepatientinnen, weshalb zur Reduktion der Strahlendosis in der Darstellung der Frontalebene der p.‑a.-Strahlengang gewählt werden sollte.

#### Was werten Sie auf dem Röntgenbild aus?

##### Bestimmung des Cobb-Winkels

**Figure 1:**
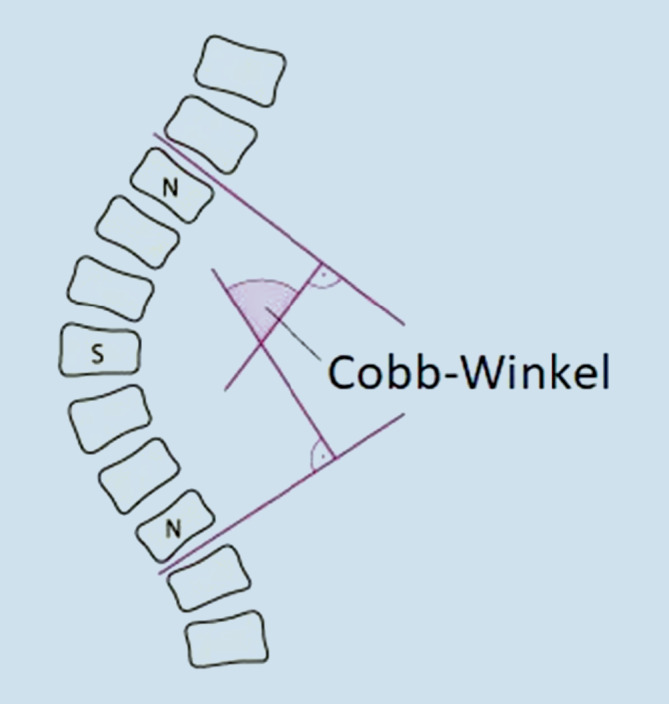

- Bestimmung des Scheitelwirbels: Er liegt im Scheitel der Hauptkrümmung (d. h. ausgeprägteste Abweichung der Wirbelsäule vom Lot) und weist die geringste Abweichung der Wirbelkörper innerhalb der Krümmung aus der Horizontalebene auf (d. h. er steht relativ horizontal), unterliegt aber der stärksten Wirbelrotation. Dies ist an den projizierten Wirbelbogenansätzen zu erkennen.
- Bestimmung der Neutralwirbel: Diese bilden den Übergang von der Hauptkrümmung zu den Nebenkrümmungen (diese können als Kompensation zur Hauptkrümmung nichtstrukturell sein, d. h. sie weisen keine wesentliche Rotation der Wirbelkörper auf). Sie weisen die stärkste Abweichung aus der Horizontalebene (d. h. sie stehen meist sehr schief), aber die geringste Wirbelrotation auf.
- Der zwischen der Deckplatte des kranial gelegenen und der Grundplatte des kaudal gelegenen Neutralwirbels liegende Winkel wird als Cobb-Winkel bezeichnet. Er ist ein Maß für die Ausprägung der Skoliose (Abb. 1).

##### Bestimmung der Rotation des Wirbelkörpers nach Nash/Moe.

**Figure 2:**
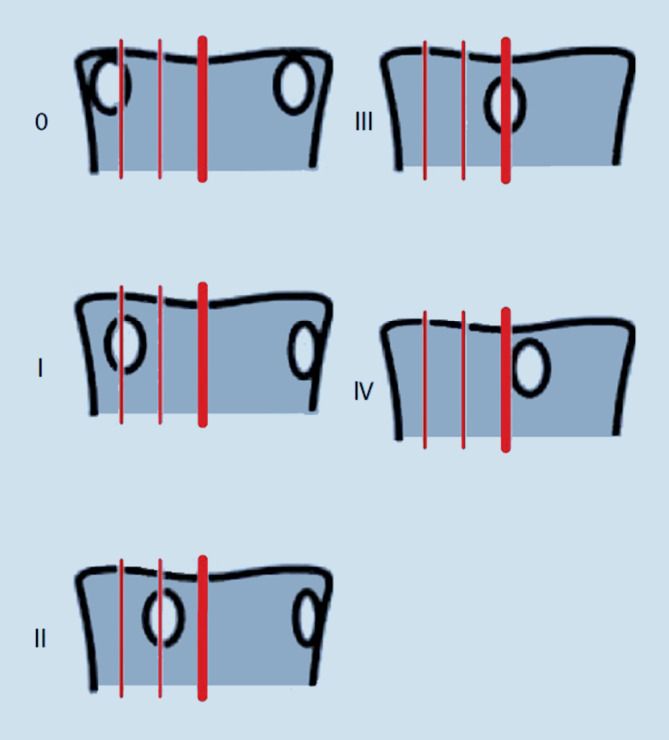

Hierfür erfolgt eine Beurteilung der projizierten Pedikel (Abb. 2).

##### Bestimmung spezieller Werte.

Spezialwissen vor geplanter OP!

- „lumbar spine modifiers“
  - Dieser Parameter wird bestimmt, um die Veränderungen im lumbalen Bereich der Skoliose zu erfassen.
  - Es werden 3 Typen unterschieden (A, B, C). Auf der a.-p.-Ebene des Röntgenbildes wird eine senkrechte Linie über die Mitte des Sakrums nach kranial gelegt.
  - Als nächster Schritt erfolgt die Bestimmung des stabilen Wirbels, welcher durch die Linie in nahezu 2 gleiche Teile geteilt wird, sollte dies auf Ebenen einer Bandscheibe sein, wird der darunterliegende Wirbelkörper als stabiler Wirbel bezeichnet.
- „sagittal-thoracic-modifiers“
  - Hierfür erfolgt die Messung der Kyphose nach Cobb zwischen Th5 und Th12 im seitlichen Röntgenbild.
  - Die Messergebnisse werden mit „−“, „N“ oder „+“ festgehalten. Als „N“ wird eine Kyphose zwischen 10° und 40° bezeichnet. Werte darüber oder darunter werden dementsprechend mit „−“ oder „+“ hinterlegt.

##### Bestimmung des Risser-Zeichens.

**Figure 3:**
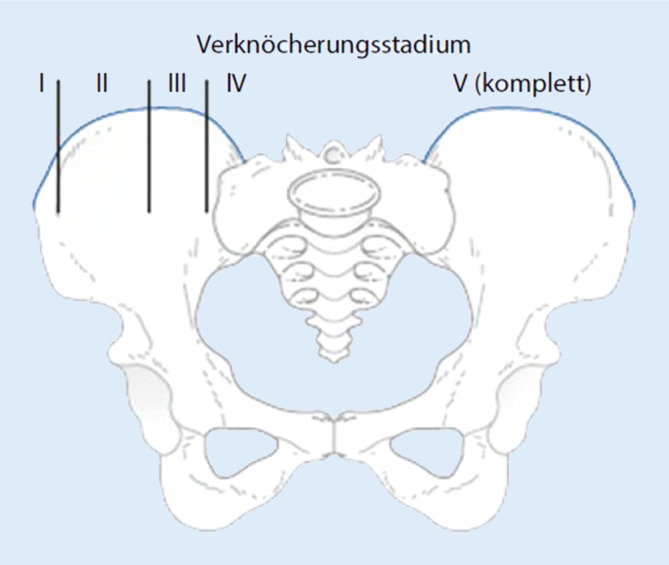
- Heutzutage kommen neben den Risser-Stadien noch weitere Klassifikationen zur Abschätzung des Restwachstums der Wirbelsäule zum Einsatz.

- Mit dem Risser-Zeichen bestimmt man die Verknöcherung der Beckenkammapophysen. Die verschiedenen Stadien des Risser-Zeichens geben Rückschlüsse auf die Knochenreife und somit auf das zu erwartende Skelettwachstum. Eine Einteilung erfolgt in Deutschland von Stadium 0 bis Stadium 5 (Abb. 3). Entscheidend hierbei ist, dass ab einem Risser-Stadium 3–4 keine wachstumsbedingte Korrektur der Wirbelsäule durch eine Korsetttherapie mehr möglich ist, da das Wachstum im Normalfall bereits abgeschlossen ist. Es ist ein klarer Indikator dafür, ob eine Korsettbehandlung notwendig oder noch sinnvoll ist.

##### Der Fall.

**Figure 4:**
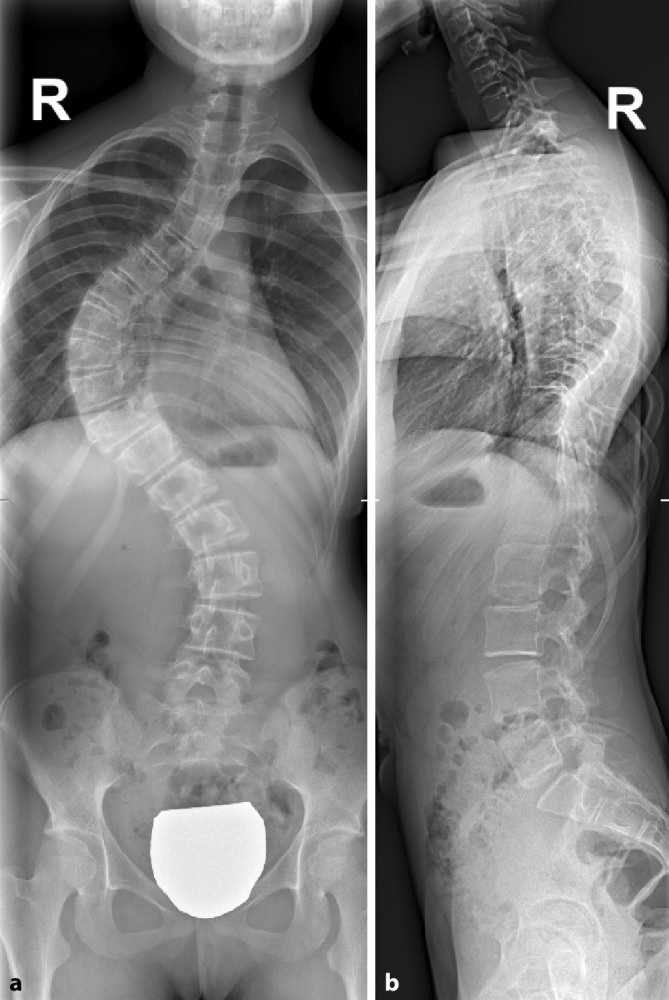

Der thorakale Cobb-Winkel von Th5 bis Th12 beträgt ca. 80° im Sinne einer strukturellen Krümmung und der lumbale Cobb-Winkel von Th12 bis L4 beträgt ca. 43° im Sinne einer kompensatorischen, nichtstrukturellen (mit praktisch neutraler Rotation) Krümmung. Eine Rotation der Wirbelkörper Grad 2 nach Nash/Moe zeigt sich in der thorakalen und Grad 1 in der lumbalen Krümmung. Der „sagittal-thoracic-modifier“ ist N bei einer Kyphose von ca. 20° von Th5 bis Th12. Darstellung des Risser-Zeichens Stadium 3 (Abb. 4).

#### Nach welchem Klassifikationssystem erfolgt die Einteilung der idiopathischen Skoliose?

- Die heutige Standardeinteilung der idiopathischen Skoliose erfolgt nach **Lenke** und wurde im Jahr 2001 eingeführt [2]. Als Basis der Einteilung dienen Röntgenaufnahmen der gesamten Wirbelsäule in 2 Ebenen und Bendingaufnahmen nach rechts und links. Anhand der zu bestimmenden Parameter lassen sich insgesamt 42 Untertypen der idiopathischen Skoliose bestimmen.
- Vor der Einführung der Klassifikation nach Lenke erfolgte eine Einteilung nach King, welche aktuell kaum noch verwendet wird.

##### Der Fall.

Durch die Röntgenaufnahmen der gesamten Wirbelsäule in 2 Ebenen sowie den Bendingaufnahmen kann die idiopathische Skoliose als Lenke 1B eingeteilt werden.

#### Welche Therapie leiten Sie ein und nach welchen Kriterien bestimmen Sie die Therapie?

Grundsätzlich gibt es die Möglichkeit der konservativen und der operativen Therapie. Anhand der radiologisch festgelegten Kriterien entscheidet letztlich der Cobb-Winkel über die Art der Therapie.

- Bis zu einem **Cobb Winkel von 10°** ist primär keine Therapie notwendig, eine radiologische Verlaufskontrolle sollte jedoch stattfinden.
- Bis zu einem **Cobb Winkel von 20°** wird die Durchführung einer regelmäßigen und spezifischen Physiotherapie empfohlen. In diesem Zusammenhang ist die Schroth-Therapie zu nennen, da sie in Deutschland am weitesten verbreitet ist.
- Bei einem **Cobb-Winkel von 20–40°** wird die Therapie um eine begleitende Korsettbehandlung erweitert. Dabei kommt in der Regel ein Cheneau-Korsett zum Einsatz, welches mindestens 16 h pro Tag getragen werden sollte.
  - NB: Die Empfehlung der 23-stündigen Tragedauer des Korsetts basiert auf theoretischen Überlegungen und wird in der Praxis seltenst eingehalten, weshalb sie als absolute Forderung unrealistisch erscheint.
- Ab einem **Cobb-Winkel von 40° lumbal und/oder 50° thorakal** wird eine operative Strategie empfohlen.

##### Der Fall.

Bei der Patientin zeigt sich in den Röntgenaufnahmen ein Cobb-Winkel thorakal von mehr als 50° und lumbal von mehr als 40°. Zudem befindet sich die Knochenreife nach Risser im Stadium 3–4.

**Figure 5:**
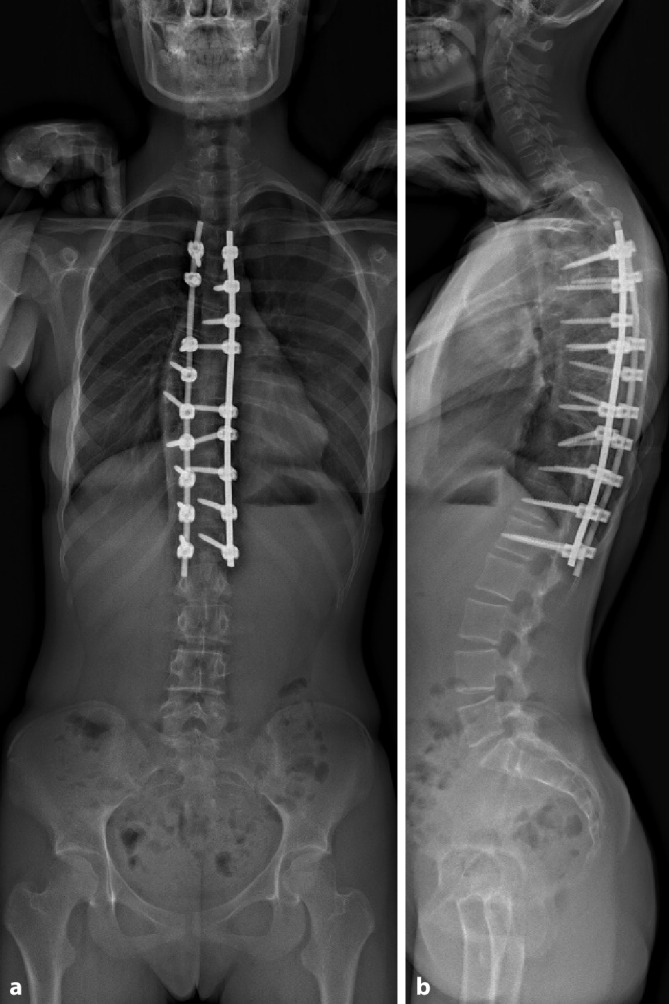

Die Kriterien für eine konservative Strategie treffen in diesem Fall nicht mehr zu und somit ist ein operatives Verfahren zu empfehlen (Abb. 5).

#### Wann würden Sie die Strategie einer konservativen Therapie ändern?

Die konservative Therapie ist abhängig von der Skelettreife, dem Cobb-Winkel, der Flexibilität der Krümmungen sowie deren radiologisch nachgewiesenen Progredienz und der Compliance des Patienten. Ein Strategiewechsel, sollte dann durchgeführt werden, wenn eine **Progredienz der Krümmung** trotz regelrecht durchgeführter Therapie nachgewiesen werden kann. Es ist zwingend erforderlich, die Compliance des Patienten zu beurteilen hinsichtlich der durchgeführten Physiotherapie und der Tragezeit des Korsetts.

#### Nennen Sie 2 weitere Formen von Skoliosen.

- **Neuromyopathische Skoliosen**, bedingt durch Nerven- und Muskelerkrankungen wie zum Beispiel spinale Muskelatrophien, Poliomyelitis und Zerebralparesen.
- **Kongenitale Skoliosen**, bedingt durch Wirbelkörperfehlbildungen.
